# Population specific reference ranges of CD3, CD4 and CD8 lymphocyte subsets among healthy Kenyans

**DOI:** 10.1186/1742-6405-10-24

**Published:** 2013-11-07

**Authors:** Erick M Bosire, Anthony K Nyamache, Michael M Gicheru, Samoel A Khamadi, Raphael W Lihana, Vincent Okoth

**Affiliations:** 1 Department of Zoological Science, Kenyatta University, P.O. Box 43844-0100, Nairobi, Kenya; 2 Department of Plant and Microbial Sciences, Kenyatta University, P. O. Box 43844-0100, Nairobi, Kenya; 3 Kenya Medical Research Institute, Centre for Viral Research, P.O Box 54840-00200, Nairobi, Kenya

**Keywords:** CD4/CD8 count, Lymphocyte subsets, HIV-1, Flow cytometry, Reference ranges

## Abstract

**Background:**

The enumeration of absolute CD4 counts is of primary importance for many medical conditions especially HIV infection where therapeutic initiation depends on the count. These ranges tend to vary across populations. However, these ranges have not been comprehensively established in the Kenyan population. Therefore, this study aimed at establishing the reference ranges for the CD4 and CD8 T-lymphocytes in normal healthy individuals in Kenya.

**Methods:**

A total of 315 individuals of the ages between 16 and 60 years old, in 5 different regions of the country, were recruited into the study. They were screened for diseases that potentially cause lymphocyte homeostasis perturbation. CD4/CD8 Counts were performed by use of a FACSCalibur flow cytometer (Becton-Dickinson, NJ) equipped with automated acquisition and analysis software. Results were analysed according to age, sex and region.

**Results:**

Results were presented as means and ranges (in parenthesis) generated non parametrically as 2.5 and 97.5 percentiles as follows; In general population; CD3 1655 (614-2685 cells/μL ), CD4 920 (343-1493 cells/μL), and CD8 646 (187-1139 cells/μL), while according to sex, females; CD3 1787 (697-2841 cells/μL), CD4 1010 (422-1572 cells/μL), CD8 659 (187-1180 cells/μL); males; CD3 1610 (581-2641 cells/μL), CD4 889(320-1459 cells/μL) and CD8 644 (185-1140 cells/μL). The general reference ranges for CD4/CD8 ratios were as follows; general population 1.57(0.50-2.74), males 1.51(0.49-2.64) and females 1.69(0.55-2.95).

**Conclusion:**

The lymphocyte reference ranges for the Kenyan population are fairly comparable to those established in other African populations. The ranges also differ appreciably from those established in Germany, Italy and Switzerland. Furthermore, the study reported significant differences in the ranges of different population clusters within Kenya, as well us between males and females.

## Introduction

Lymphocytes co-ordinate the immune system’s response and play a central role in cell mediated immunity [1]. Lymphocyte subset may include; Helper T cells (CD4 T-cells), Cytotoxic T cells (CTLs or CD8 T-cells), Memory T cells and Regulatory T cells (Treg cells). Upon encounter with antigens, CD4 T cells become activated and proliferate rapidly secreting cytokines that sends signals and maintain active immune response. On the other hand, the CD8 T-cells destroy virally infected cells and tumor cells, and remain inactivated when there is no foreign antigen. In human immunodeficiency virus-1 (HIV-1) infected individuals, lymphocytes (specifically CD4 T-cells) are the viral prime targets. Therefore in these individuals, a CD4 count provides a picture of immune system competence, with higher CD4 counts typically signifying healthier immune systems [1,2]. In such application, flow cytometry is used to provide absolute counts, percentages and or ratios of these lymphocyte subsets. Besides HIV management, flow cytometric analysis of peripheral blood lymphocyte phenotypes has proven a useful aid in managing a wide range of medical conditions, including autoimmunity, immunodeficiency, infection, malignancy and transplantation [3-5]. Essential to the effective application of this approach is availability of accurate reference values against which results can be meaningfully compared.

Reference ranges that are currently used in Kenya are derived from data obtained from Caucasians who are not African Kenyans. In addition, these ranges do not include age and sex which are very important factors that influence lymphocyte counts [6]. Furthermore, several factors have been associated with these differences in lymphocyte counts. These may include; demographic and genetic factors, current exposure to infectious diseases and behavioral factors on CD3, CD4 and CD8 in HIV-negative populations [1,2,6]. Averagely, healthy African and Asian populations have been shown to have lower CD4 lymphocyte counts than their western European and Caucasian counterparts [2,7,8]. In addition, women tend to have higher CD4 levels than men with comparable demographic and behavioral patterns [8,9]. However, there is still limited data from specific countries to confirm these differences.

The pattern of lymphocyte generation has been shown to affect the levels of circulating lymphocytes in males, females and individuals of different ages. The pattern of T lymphocyte generation in aging has been associated with dynamic changes in thymic and extrathymic functions along with sequential developmental steps from cells to mature cells [10]. Increased absolute numbers of peripheral blood CD4 cells in females compared to males have been reported [11]. This is perhaps due to androgens which accelerate thymocytes apoptosis and subsequently influence T cell repertoire with males tending to have less CD4 cells than females [12,13]. Sex and age related variations in the lymphocyte subsets contribute to age and sex related diseases including autoimmune disorders in females and leukemia or lymphoma in males and elderly patients [14,15]. Other factors which have received little attention but also affect the normal lymphocyte levels include; dietary patterns, body mass index and smoking habits [16-18]. The influence of these many factors point out to the fact that the reference ranges of one population might not be accurately used as a reference range for another. This might give an inaccurate interpretation of the immune status of the individuals.

Several efforts to establish reference ranges have indicated differences in lymphocyte levels between African and Western populations [2,3,9,19]. In one community in Kenya, general clinical ranges have been shown to significantly differ from the ranges from other parts of the continent [20]. These ranges are also appreciably different from those reported in a neighboring country [21]. The two studies in Kenya had indicated a possibility of having remarkably different ranges of lymphocyte subsets within the Kenyan population [20,22]. This necessitates the need for a comprehensive reference range establishment using subjects from different regions of the country. In this study we report comprehensive reference ranges for CD3, CD4 and CD8 cutting across representative regions of the country. The study provides ranges specific for males, females and for different ages.

## Materials and methods

### Study subjects and samples

A total of 315 adults (222 men and 85 female) were recruited into the study after giving consent. This study was ethically approved by Kenya National ethical Review committee before execution. The individuals who were included in the study were healthy adults between 16 and 55 (categorized as 16-19, 21-30 and 31-55) years of age presenting for routine blood donation at the regional centers. These regional blood transfusion centers included; Mombasa, Nakuru, Kisumu, Embu, and Nairobi. A structured questionnaire was used to collect information on the history and general health status of the donors (See Additional file 1). Donors who confirmed to have a history of conditions that cause immune perturbations were excluded from the study. Further, the samples were screened for HIV, Hepatitis B virus (HBV), Hepatitis C virus (HCV), malaria, and syphilis after blood collection, and samples that tested positive were excluded from the study. Blood samples were collected by venipuncture into EDTA vacutainer tubes and analyzed immediately after collection.

### Screening for malaria, syphilis, HIV, HCV and HBV

Enzyme linked immunoassays were used in screening for these conditions. Commercially available kits containing purified antigens were used. The kits included: ICE* Syphilis, Murex anti-HCV (version 4.0), Hepanostika HBsAg Ultra kit, Murex HIV-1.2.0 (Abbot) and malaria antigen detection kit (Creative diagnostics).

### Sample collection and analysis

The lymphocyte subsets were analyzed on a FACSCalibur flow cytometer (Becton Dickinson Immunocytometry Systems, San Jose, California) with three monoclonal antibodies (BD Tritest CD4-FITC/CD8-PE/CD3-PerCP reagent) according to the manufacturer’s instructions. The FACSCaliber was calibrated and reconfirmed daily using the BD Calibrite beads and BD-FACSComp software, version 2.0 for setting the photomultiplier tube voltages, setting the fluorescence compensation and checking instrument sensitivity before use. A control sample of commercially available whole blood was run daily to optimize the instrument settings. The CD45 versus the side scatter (SSC) dot plot was visually inspected to make sure they appear as a bright compact cluster, with low SSC. Analysis did not proceed if populations were diffuse and if there was little or no separation between clusters [15].

### Data analysis

Non parametric reference range determination was employed by estimating the 2.5 and 97.5 percentile [23]. The significance of differences between the mean absolute counts and percentages of the Lymphocyte subsets were estimated using the Analysis of Variance (ANOVA) and T-test. Multiple comparisons were done using Tukey’s test. Correlation analysis was applied in determining the relationship between lymphocyte subset levels and age.

## Results

Among the 315 individuals between the ages 16-60 years, 61.7% (222) were men and 38.4% (85) were female. Their blood samples were analyzed for CD3, CD4 and CD8 lymphocyte subset for establishment of reference ranges. These reference ranges were also compared across the sampled geographical regions, by age as well as sex.

### General distribution of the T-lymphocytes for the study population

The CD3, CD4 and CD8 counts and ranges for the general study population were determined. The means, median, percentages and ranges of these lymphocytes, as well as the CD4/CD8 ratios were determined (Table 1).

**Table 1:** The means and medians of the lymphocyte subsets in the study population

**T lymphocyte subset**							
	**CD3**		**CD4**		**CD8**		**Ratios**
	Absolute count^*^	**%**^**×**^	Absolute count^*^	**%**^**×**^	Absolute count^*^	**%**^**×**^	**CD4/CD8**
**Mean**	1655	67	920	37	646	26	1.57
**Median**	1580	67	884	37	559	25	1.47
**Ranges**	614-2685	50-78	343-1493	24-48	187-1139	13-36	0.50-2.74

^*^Expressed as cells/μL of blood.

^**×**^Expressed as a percentage of total lymphocytes.

### Lymphocyte subsets in the different Kenyan regions

Mean absolute counts of the different regions were compared using ANOVA. Significantly different means were further subjected to Tukey’s test to show differences between each of the regions. The difference in the mean CD3 of Nairobi and Rift valley regions was minimal compared to the difference in the CD3 mean of the Lake Victoria basin region. The Lake Victoria region had the lowest CD3 mean compared to all other regions. These differences in the CD3 absolute count means were significant. General reference ranges were also determined as mentioned earlier (P < 0.05; Table 2).

**Table 2:** Means, medians and ranges of the lymphocyte subsets for the different geographical regions in Kenya

**Region**	**Lymphocyte subset**						
		**CD3 count**	**CD3%**	**CD4 count**	**CD4%**	**CD8 count**	**CD8%**
**Nairobi**	Mean ± SD	1841 ± 486c	69 ± 6a	1046 ± 283c	39 ± 6a	717 ± 260c	26 ± 5c
	Median	1824	69	1007	39	691	26
	Range	908-2661	55-79	517-1506	27-48	266-1165	16-35
**Eastern**	Mean ± SD	1728 ± 642b	64 ± 7a	949 ± 209b	37 ± 7b	650 ± 329ab	24 ± 6ab
	Median	1617	64	959	37	529	22
	Range	813-2512	47-77	512-1320	21-50	213-1083	11-34
**Rift valley**	Mean ± SD	1876 ± 883d	67 ± 8b	1029 ± 559ab	37 ± 7c	752 ± 421d	27 ± 8b
	Median	1666	68	902	36	640	26
	Range	660-3074	50-80	345-1703	23-47	217-1315	13-39
**Lake Victoria**	Mean ± SD	1195 ± 449abcd	66 ± 7c	690 ± 263abc	38 ± 6d	454 ± 231acd	25 ± 7d
	Median	1137	67	630	38	401	24
	Range	471-1886	50-77	275-1081	26-47	135-791	12-36
**Coast**	Mean ± SD	1617 ± 574a	66 ± 7d	881 ± 333a	36 ± 7a	675 ± 289a	28 ± 6a
	Median	1502	67	854	36	602	28
	Range	572-2708	51-77	281-1540	22-47	210-1179	16-37

The absolute counts of CD4, CD3 and CD8 are presented as cells/μL of blood.

Means followed by the same letters within a column are not significantly different from each other according to Tukey’s test.

### T-lymphocyte subset for males and females

Females had a significantly higher CD3 mean absolute counts (P < 0.05) compared to men. There were significant differences in CD4 counts for males and females. However, there were no significant differences in the mean CD8 absolute counts. T lymphocyte subset reference ranges for males and females separately were also determined at 2.5 and 97.5 percentiles (Table 3).

**Table 3:** Means, medians and ranges of the lymphocyte subsets for males and females

**Lymphocyte subset**	**Sex**		**p-value**
	**Female**	**Male**	
CD3 Absolute count^*^			
N	85	222	
Mean	1787	1610	0.039
Median	1701	1529	0.016
Ranges	697-2841	581-2641	
**CD3% ˟**			
Mean	68	66	0.026
Median	69	66	0.024
Ranges	54-78	49-78	
CD8 Absolute count^*^			
Mean	659	644	0.743
Median	568	558	0.696
Ranges	187-1180	185-1140	
**CD8%**^**×**^			
Mean	25	26	0.133
Median	25	25	0.256
Ranges	14-34	13-37	
CD4 Absolute count^*^			
Mean	1010	889	0.011
Median	991	855	0.001
Ranges	422-1572	320-1459	
**CD4%**^**×**^			
Mean	40	37	0.001
Median	39	36	0.001
Ranges	23-53	24-46	
**CD4/CD8 ratio**			
Mean	1.69	1.51	<0.001
Median	1.62	1.44	0.030
Ranges	0.55-2.95	0.49-2.64	

^*^Expressed as cells/μL of blood.

^**×**^Expressed as a percentage of total lymphocytes.

### T-lymphocyte subset for the different age groups

The means and medians of the lymphocyte subsets were also determined for the different age groups. There were no significant differences in the mean absolute counts in the age groups that were considered in this study (Table 4).

**Table 4:** The lymphocyte subset levels in the different age groups

**Subset**	**Age group**			
	**16-19**	**20-30**	**31-55**	**P value**
CD3 Absolute count*				
N	55	196	62	
Mean	1603	1641	1742	0.745
Median	1519	1611	1622	0.595
**CD3%**^**×**^				
N	55	196	62	
Mean	65	67	67	0.180
Median	65	67	67	0.261
CD8 Absolute count^*^				
N	52	181	61	
Mean	611	641	693	0.870
Median	526	564	636	0.498
**CD8%**^**×**^				
N	52	181	61	
Mean	24	26	27	0.138
Median	23	25	26.	0.090
CD4 Absolute count^*^				
N	55	196	62	
Mean	912	898	997	0.139
Median	915	882	988	0.254
**CD4%**^**×**^				
N	55	196	62	
Mean	38	37	38	0.222
Median	36	37	37	0.184
**CD4/CD8 ratio**				
Mean	1.70	1.54	1.54	<0.001
Median	1.63	1.45	1.51	0.135

^*^Expressed as cells/μL of blood.

^**×**^Expressed as a percentage of total lymphocytes.

ANOVA was used to test the differences in means of the lymphocytes in the age groups.

### Comparison of the ranges of the current study with those established by other studies

The reference ranges established in this study showed variations with the ones used in MultiSET (BD FACScaliber) as well as other studies in different populations. The studies compared below included normal (HIV negative) populations in their study populations. Most of the studies were regional, that is, having been conducted in specific countries or in some specific regions within the countries (Table 5).

**Table 5:** Comparison of the reference ranges developed in this study with reference ranges of other populations

**Subset**	**Kenya**	**MultiSET**	**Tanzania**	**Ghana**	**Italy**	**Germany**	**Switzerland**
CD3^*^	613.9048-2685.439	690-2540	NA	NA	600-2460	780-2240	540-1790
CD3%^**×**^	50.3433-78.0606	55-84	NA	NA	NA	NA	NA
CD4^*^	343.5796-1493.167	410-1590	312.2-1367.6	265-1932	490-1670	490-1640	310-1140
CD4%^**×**^	23.7048-48.2465	31-60	NA	NA	NA	NA	NA
CD8^*^	187.1348-1139.389	190-1140	168.2-996.8	162-1590	220-1110	170-880	140-820
CD8%^**×**^	13.3894-36.3925	13-41	NA	NA	NA	NA	NA
CD4/CD8 ratio	0.5002-2.7434	NA	1.1-2.5	0.58-4.59	NA	0.9-5.0	1.0-5.0

^NA^Not available.

^*^Expressed as cells/μL of blood.

^**×**^Expressed as a percentage of total lymphocytes.

Kenya-Current study, MultiSET-BD Biosciences, Tanzania [19], Ghana [2], Italy [24], Germany [25], Switzerland [3].

## Discussion

The reference values of the main circulating lymphocyte subsets have been established by many studies throughout the world and have shown some variability according to geographical locations and methodology. Results in Table 5 show comparative values of CD3, CD4 and CD8 subsets levels from other countries, showing variations based on geographical region and platform methods used.

In this study, the mean CD3 count was found to be generally higher compared to those reported in Sweden (1075) but lower in CD3% mean [3]. In comparison with other African countries the lymphocyte counts and percentages were found to be fairly comparable in some cases but in others slightly different. In addition, Kenya like many African countries (Tanzania, Ghana, Senegal and Ethiopia) had lower lymphocyte subset levels compared to those reported in Sweden [3,25,26].

Considering the two important lymphocyte subsets CD4 and CD8, there were striking variations with the other reported ranges within and outside Africa. The mean CD8 value for this study was similar to those obtained in other African countries but slightly higher than those reported in Tanzania and Senegal [8,25]. The CD4 absolute counts were significantly higher than those reported in Ethiopia and Senegal [17] but lower compared to those reported in Kampala Uganda [21]; Table 5. However, the ranges cannot be conclusively compared since the studies differ in experimental set up. For example the study in Uganda included patients who visited an AIDS information center in Kampala, whereas our study was among blood donors across the country. In comparison to other countries outside Africa, the CD4 absolute counts were higher compared to Asian populations [27]. It can be speculated that, these differences in the lymphocyte counts are due to the many factors already mentioned, including genetic predisposition [1-3]. Many of these factors including the genetic influence have not been comprehensively confirmed.

In this study, females had higher absolute counts for CD3 and CD4 lymphocytes compared to males. This was probably due to biological factors (Table 4). It has been speculated that gender and age-related variations within the immune system parameters may contribute to the pathogenesis of several gender and age-related diseases such as autoimmune disorders in female patients [17,19,24]. Further, it has been shown that there are gender differences in the generation of CD8 cells during HIV-1 infection, due to increased immune activation compared to men [28]. However, a comparison of mean CD8 absolute counts between males and females, revealed no significant differences. These findings were consistent with previous studies [2,3,19,24].

The study also compared the differences in absolutes counts across regions. There were significant differences across regions with Lake Victoria region, consistently recording lower lymphocyte counts compared to other regions. The CD8 absolute count of Nairobi were appreciably similar with ones reported by Embree [19] in their controls of a study carried out in Nairobi. These observations show that, there is need to understand the impact of genetic makeup and altitude on the levels of T lymphocytes. The CD4 median absolute counts for the Rift valley (810 cells/μL) were similar to the ones reported in one of the studies in Kericho which is located within the valley ([20] Table 2).

Analysis of the lymphocyte subsets in the different age groups revealed no significant differences. A more comprehensive study design might be required to give a clear picture of the lymphocyte changes with advancing age. It is however well known that the pattern of T lymphocyte generation with age originates from dynamic changes in thymic as well as extrathymic functions, along with sequential developmental steps from stem cell to ultimately mature cells [29].

## Conclusion

Our study has established ranges and reveals that lymphocyte subsets among the Kenyan population are different from those of other populations in the other populations such as Germany, Italy and Switzerland. However, the ranges are fairly comparable to those reported in some African countries. The ranges are also slightly different from those that are being used currently. This study has established reference ranges for the lymphocyte subsets among healthy Kenyan individuals of the ages between 16-60 years using FACSCalibur flow cytometer (Becton Dickinson Immunocytometry Systems, San Jose, California). The study has further shown the possibility of having varied lymphocyte levels among Kenyan subpopulations, due to many unconfirmed factors. Therefore, the current ranges should be more comprehensively determined and further revised, in order to have accurate comparisons during flow cytometry.

## Abbreviations

HIV: Human immunodefieciency virus; CTL: Cytotoxic T lymphocytes; SSC: Side scatter; ANOVA: Analysis of variance.

## Competing interests

The authors declare that they have no competing interests.

## Authors’ contributions

EB designed the study, collected samples, analysed them and drafted the manuscript. MMG helped in data analysis and interpretation and reviewed the manuscript. SAK conceptualized the idea, helped in designing experiment, analysis of the samples, interpretation of data and review of the manuscript. AKN helped in drafting and critically revising the manuscript. RL helped in critically reviewing the manuscript. VO helped in critical review of the paper. All authors read and approved the final manuscript.

## Supplementary Material

Additional file 1 Questionnaire for sample collection. Click here for file
