# Supplementary material for: Population specific reference ranges of CD3, CD4 and CD8 lymphocyte subsets among healthy Kenyans
Source: AIDS Res Ther. 2013 Nov 7;10:24. doi: 10.1186/1742-6405-10-24 (PMC3827884; doi:10.1186/1742-6405-10-24)
Supplement: Additional file 1 — Questionnaire for sample collection. [file 1742-6405-10-24-S1.docx]

**Additional File**

**Additional file 1: Questionnaire for sample collection**

The information will be treated confidentially.

Subject ID…………..

Age…………..

Sex…………...

Location……….

1. Indicate your state for the following

|  |
| --- |
|  |
|  |
|  |
|  |
|  |
|  |
|  |
|  |
|  |
|  |

1. Transfusion in the last one year
2. Donated blood in the last four months
3. Major operation in the last 2 years
4. Dental work in the last 1 week
5. Body piercing in the last 1 year
6. Currently under medication or unwell
7. Multiple sex partners
8. Not sure about my sexual partner
9. Sexual contact with a suspicious partner
10. STD infection in the last 1 year
11. Pregnant or breastfeeding
12. Do you have any of the following conditions

(Tick where applicable)

|  |
| --- |
|  |
|  |
|  |
|  |
|  |
|  |

1. Anaemia or bleeding disorder
2. Diabetes
3. Epilepsy
4. Heart disease
5. Stomach ulcers
6. High Blood Pressure
7. Jaundice
